# Observations on the Juice of the Papaya

**Published:** 1803-01-01

**Authors:** 


### *Observations on the Juice of the Papaya;* *by* Cit. VAUQUELIN.

IT is related by Cit. Charpentier de Cossigny, that the juice of the Papaya, (*Caraca Papaya, L.*) is successfully employed in the isle of Bourbon against the tape-worm, on which account Cit. VAUQUELIN reduced it to a chemical analysis. From this it appears, that the juice, dried without any previous preparation, puffs up on burning coals, emitting an odour like that of burning flesh; it yields a great great quantity of ashes, which afford before the tube a phosphorescent light, and consist of pure phosphat of lime. The dry juice of the Papaya easily dissolves in water, to which it imparts a milky colour, owing to a substance which is not soluble. If suffered to stand, the water becomes clear; but soon after it turns putrid, diffusing a very offensive smell. The insoluble substance being collected, had all the characteristics of an animal fat. Nitric acid makes a precipitation in the solution of the juice of the Papaya, and the precipitated matter takes the consistency of a jelly. If the solution be previously boiled, white flakes fall to the bottom, and it is not capable of any farther precipitation by nitric acid, but only by an infusion of galls. Alcohol occasions likewise a precipitation in the solution of the juice of the Papaya. It is, however, soluble in alkalis; and when decomposed by acids, it emits a nauseous smell. From these and other characters, it appears to bear a very great similitude to the serum of the blood, and perhaps to the blood itself, particularly as Cit. Vauquelin thinks he has observed traces of a fibrous part in the insoluble part of the juice. Cit. de Cossigny has brought with him an extract of the Papaya, which is half transparent, of a reddish colour, and prepared by evaporating a solution of the juice of that tree in rum. This extract, on being submitted to the same experiments as the dry juice, caused some difference to appear. Its taste was similar to that of raw meat, and it was not coagulated by heat; acids had no effect upon it. Vauquelin compares it with animal gelatine; and he thinks that it has acquired these properties by its solution in rum, and by its having been evaporated to the consistency of an extract.

Cit. Vauquelin terminates his notice with observing, that there is a singular analogy between this juice and a certain animal liquor; and he mentions, that Fourcroy has already discovered traces of albuminous matter in the juices of some plants; and that Scheele mentions his having found in the leaves of vegetables, a sort of caseous substance; moreover, Cit. Proust asserts, that the milk of sweet almonds is a combination of oily and caseous particles.

